# A mechanistic study on the humic substance formation from 5-(hydroxymethyl)-furfural in alkaline solutions†

**DOI:** 10.1039/d5ra02859k

**Published:** 2025-07-16

**Authors:** Marten Niklas Gey, Uwe Schröder

**Affiliations:** a Institute of Biochemistry, University of Greifswald Felix-Hausdorff-Str. 4 17489 Greifswald Germany uwe.schroeder@uni-greifswald.de

## Abstract

One of the major challenges in the oxidation of the carbohydrate-based 5-(hydroxymethyl)-furfural (HMF) to 2,5-furandicarboxylic acid (FDCA) – a reaction of great relevance for the production of biopolymers – is the need for alkaline conditions. Many of the published oxidation systems operate at pH 13–14, at which a self-polymerization of HMF to strongly colored humic substances occurs. To date, this side reaction has only been investigated to a limited extent. This study presents a first investigation of the humic substance formation of alkaline HMF solutions upon storage over a period of more than 200 hours. A comparison of the HMF degradation with that of its benzyl analog 4-(hydroxymethyl)-benzaldehyde (HMB) showed that humic substance formation is caused by the opening of the furan ring, which subsequently induces linking of the individual HMF molecules. Parallel to this, the Cannizzaro reaction proceeds, which, in the observed concentration range of 20–100 mM, converted approx. 20% of the initial HMF. Further analysis of the humic substance formation by UV/Vis spectroscopy revealed that this process can be separated into a “build-up” phase (within the first 24 h) and an “aging” phase (after 24 h), in which the colored humic material is decolorized again due to the presence of dissolved atmospheric O_2_. Based on the solubility at different pH values, the formed humic material was classified as a mixture of humic acids and fulvic acids, while (fully insoluble) humins were not formed. Finally, FTIR spectroscopy was utilized to carry out a structural investigation of the acid-insoluble humic acid fraction.

## Abstract 2

This study investigates the formation and structural evolution of humic substances from HMF under alkaline conditions, identifying ring-opening polymerization as the key mechanism and highlighting implications for FDCA production.

## Graphical abstract

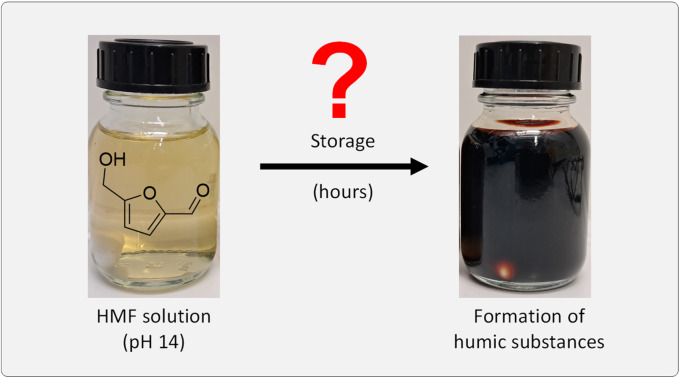

## Introduction

In the context of a sustainable materials economy, the carbohydrate-based 2,5-furandicarboxylic acid (FDCA) is an attractive substitute for petroleum-based terephthalic acid in the production of polyesters. The polymerization product of FDCA and ethylene glycol, polyethylene furanoate (PEF), can be used for the manufacturing of plastic bottles, as it possesses similar or even enhanced properties compared to polyethylene terephthalate (PET).^1,2^ FDCA can be obtained by the oxidation of 5-(hydroxymethyl)-furfural (HMF) – a dehydration product of fructose.^3,4^ Accordingly, various routes for the oxidation of HMF have been presented in the last two decades, which often use O_2_ as a green oxidant or make use of alternative catalytic methods including electrochemical or photochemical oxidations.^5–11^ Many of these oxidation processes require alkaline reaction conditions, whereby especially the latter systems typically operate in the pH range of 13–14.^10,11^ Under these conditions, however, HMF undergoes irreversible degradation reactions into humic substances – strongly colored polymers that are formed even at ambient temperature.^12,13^ This effect does not only result in a considerable loss of substance but also complicates the product clean-up, which is especially relevant as pure FDCA is needed for the production of fully transparent PEF bottles.^1,14^

The base-catalyzed humic substance (BCH) formation is an immense problem for process upscaling, *e.g.* when it comes to the storage of alkaline HMF solutions. However, in many publications on HMF oxidation under the respective conditions it is only described as an unpleasant side effect, if at all.^15–18^ While several studies have investigated the humic substance formation of HMF under acidic conditions^19–24^ – which is relevant for the production of HMF – only a few studies have so far focused on the characterization and formation mechanism of BCH. However, some fundamental information can be derived from the current literature:

(1) The BCH formation is enabled by the presence of both the aldehyde- and the alcohol functionality in the HMF molecule, which allows random linkage of the monomers. Thus, it was shown by Kim *et al.* that the formation of BCH can be prevented by acetal protection of the aldehyde group.^25^

(2) Higher concentrations of HMF and OH^−^ lead to an acceleration of the degradation reactions and thus to a higher proportion of side products.^13,26^

(3) Tashiro *et al.* carried out a computational analysis of possible oligomers formed from HMF and thus delivered an energetic assessment of the first BCH formation steps. They also investigated the effect of acetal protection on the energy levels.^27^

(4) Vuyyuru and Strasser performed time-resolved ^1^H NMR measurements of stored alkaline HMF solutions. They found that the two protons of the aromatic furan ring undergo a high-field shift (from 7.0 and 7.9 ppm to a broad signal range of 6.2–7.2 ppm) and concluded that an opening of the aromatic ring occurs during the BCH formation.^12^

(5) Multiple studies report that BCH cannot be oxidized to FDCA anymore.^16,28^ However, *e.g.* Liu *et al.* reported for their electrochemical system that BCH oxidation to FDCA was possible, without describing any details.^29^

(6) Alongside the BCH formation, some monomeric substances were found to be formed in alkaline environment. For instance, 5-(hydroxymethyl)-furancarboxilic acid (HMFCA) and 2,5-bishydroxymethylfuran (BHMF) are obtained by the Cannizzaro reaction, in which the aldehyde group of two HMF molecules are disproportionated to a carboxyl and a hydroxyl group.^26,30^ Furthermore, the formation of levulinic acid (LA) and formic acid (FA) were reported to be formed under harsh conditions (*i.e.* high temperatures and/or high oxygen or air pressure) as a result of ring opening.^30–32^

Despite this essential information, an experimentally proven formation mechanism of BCH has not been proposed so far. A general problem is that, due to the irregular structure of humic substances, it is difficult to interpret results of the conventional spectroscopic methods (*e.g.* IR or NMR). Thus, a precise analysis of the mechanism is not trivial, which can also be deduced from the ongoing discussion concerning the acid-catalyzed humic substance (ACH) formation.^19–24^

**Scheme 1:**
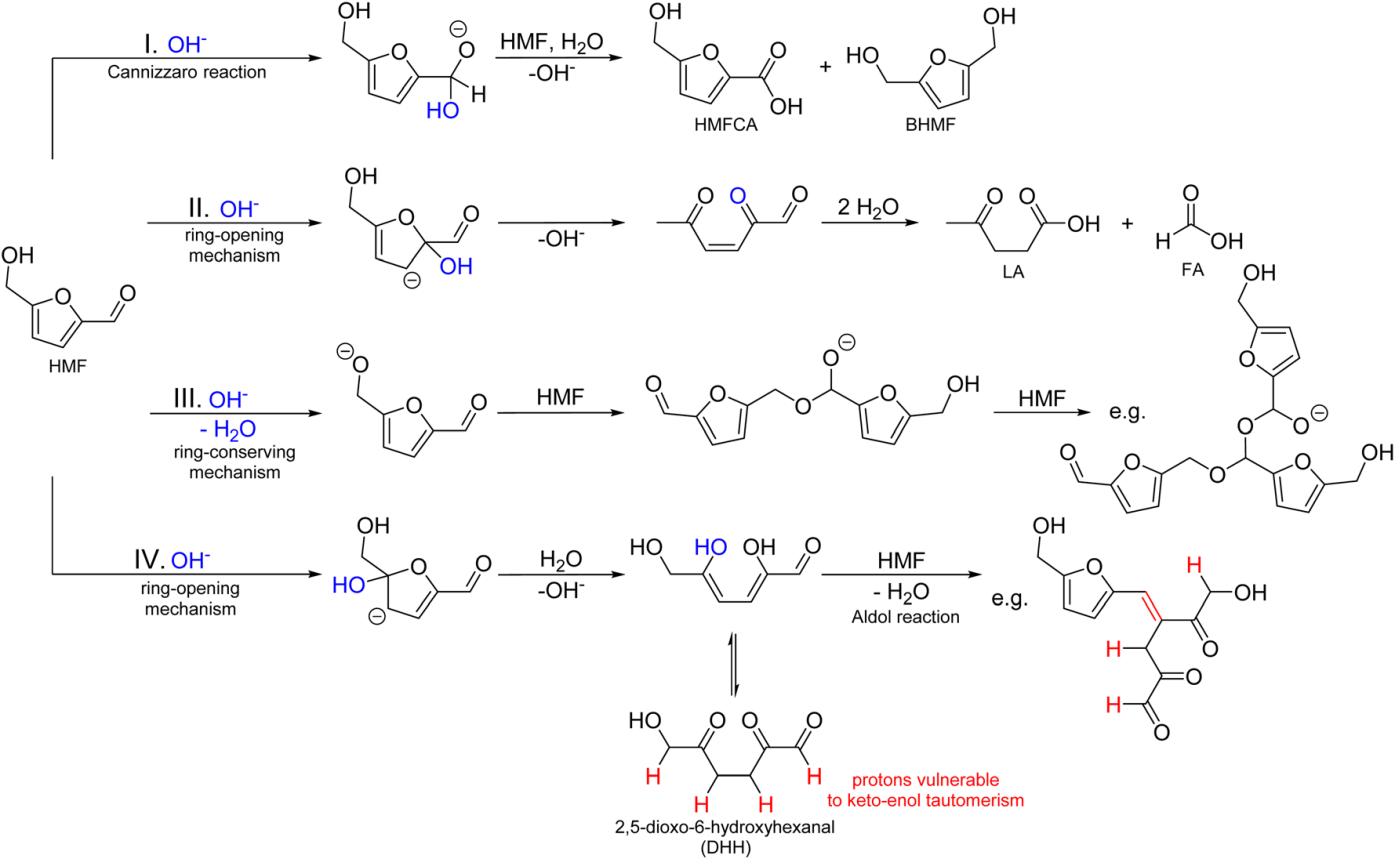
Possible mechanisms for the HMF degradation in alkaline solutions with exemplary oligomeric products.

This paper aims to provide a general overview of the topic of alkaline HMF degradation under typical storage conditions (ambient temperature, standard pressure) and to deliver a first experimental assessment of the BCH formation mechanism and structure. Based on the available information stated above and by comparison with the established mechanisms of ACH formation, a general reaction scheme can be proposed (Scheme 1).

It contains:

(I) The Cannizzaro reaction, yielding HMFCA and BHMF in a 1 : 1 ratio.

(II) The formation of LA and FA after ring-opening. This is possibly a result of a nucleophilic attack of the hydroxide ion in the 2-position of the aromatic ring, as shown in the established mechanisms for levulinic acid formation in acidic environments.^33,34^

(III) A ring-conserving polymerization mechanism. A possible reaction would be the linkage of single HMF molecules through hemiacetal formation. The reaction pathway shown corresponds to the one that leads to the most stable trimer according to the computational study of Tashiro *et al.*^27^

(IV) A ring-opening polymerization mechanism. This could proceed, for example, by the formation of 2,5-dioxo-6-hydroxyhexanal (DHH), which was first proposed for the ACH formation by Horvat *et al.*^34^ The formation of this molecule is also conceivable in alkaline solutions, as it is induced by the addition of water to a double bond of the aromatic ring. The ring-opened DHH would accordingly offer four binding positions for an aldol reaction (addition or condensation).^20,21^ However, DHH was not spectroscopically identified so far, thus suggesting a high reactivity.

It should be noted that these reaction pathways could also take place in parallel. Thus, formed products of different pathways could also react with each other to form the irregular humic structure. It is therefore crucial to identify which of the initial reaction pathways can occur.

To investigate this, we monitored the alkaline HMF degradation over a storage duration of 216 hours (9 days). HPLC analysis was used to identify the products of reactions (I) and (II) To obtain an experimental assessment of the polymerization mechanisms (reactions (III) and (IV)), we compared the degradation behavior of HMF with the analogous benzyl derivative, 4-(hydroxymethyl)-benzaldehyde (HMB). This was done because we suspected that ring-opening reactions (reactions (II) and (IV)) should not occur with HMB due to the higher stability and the lower reactivity of the benzene ring to nucleophilic attacks.^35^ Accordingly, we expected to identify if a ring-conserving polymerization mechanism (III) can occur by monitoring the HMB conversion and the production of the respective Cannizzaro products. This would allow us to make a statement whether or not a ring-opening mechanism is necessary for the BCH formation of HMF. Furthermore, we used UV/Vis spectroscopy as a simple technique to investigate the formation of the colored BCH in solution and performed a first structural analysis *via* FTIR spectroscopy.

## Results and discussion

### Alkaline degradation of HMF and HMB

To compare the degradation of HMF and HMB, solutions of both substances were dissolved in 1.0 M KOH with concentrations of 20, 50 and 100 mM and stored under stirring at RT over several days. To assess the loss of substance due to the formation of non-analyzable products (*i.e.* humic material), we calculated the mole balance, MB, for each data point. This parameter is defined as the ratio of the totalized amounts of all analyzed substances (substrate and products) to the initial amount of substrate (eqn (S3) in the ESI†).

**Fig. 1:**
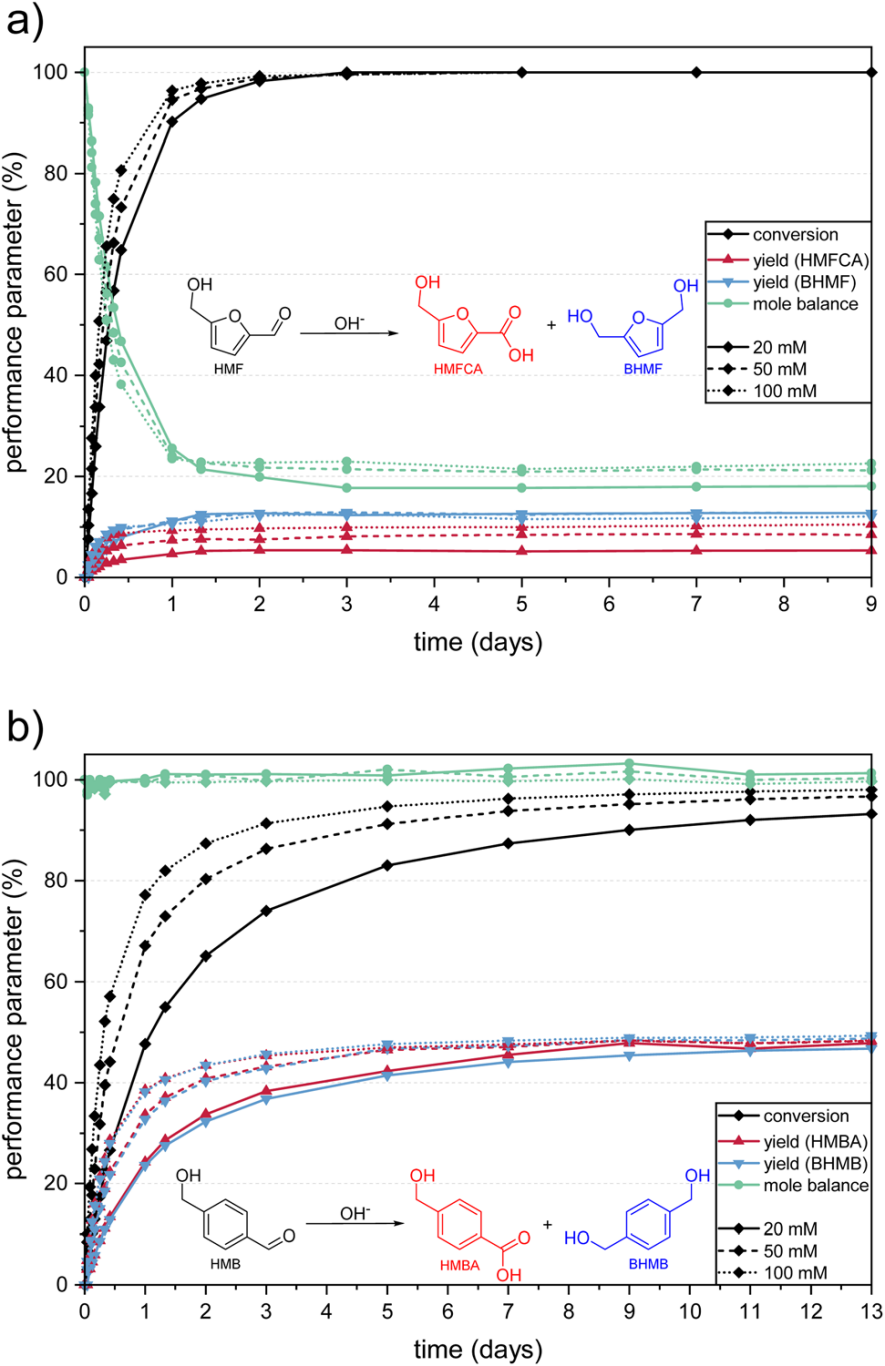
Performance parameters during the degradation of (a) HMF and (b) HMB solutions of different concentrations in 1.0 M KOH. Equations of the respective Cannizzaro reaction are given to simplify color assignment. Numerical values are stated in Table S1 in the ESI.†

A visible coloration of the light-yellow HMF solutions was already observed within the first hour after preparation, in which, depending on the initial concentration, 8–14% of the substrate was converted. After 24 h, over 90% of the HMF was depleted at all tested concentrations and almost full depletion was reached after 3 days (Fig. 1a). The product analysis showed that about 18–23% of the HMF was converted to Cannizzaro products, while LA and FA were not found in any sample. It can therefore be assumed that the base-catalyzed formation of LA and FA is only possible at elevated temperatures or oxygen pressures, as expected on the basis of the literature.^30–32^ Thus, 77–82% of the HMF was converted to humic material.

In contrast, the HMB solutions did not show any visible coloration, even after the storage time was extended to 13 days. HPLC analysis showed that the conversion was slower compared to HMF, so that no full conversion was observed in the experimental time frame (Fig. 1b). The reacting substrate was fully converted into the Cannizzaro products 4-(hydroxymethyl)-benzoic acid (HMBA) and 1,4-bis-(hydroxymethyl)-benzene (BHMB), so that MB values close to 100% were reached for the entire storage time. These results show, that a polymerization to humic substances is not possible in the absence of a furan ring, while the Cannizzaro reaction (which does not involve the aromatic ring) is still observed. Thus, it can be deduced that a ring-conserving HMF polymerization mechanism (Scheme 1, reaction (III)) can be excluded for the chosen alkaline conditions as it should also have been observed for HMB. Accordingly, it is concluded that the formation of BCH proceeds *via* a ring-opening mechanism. An aldol reaction, as shown in reaction (IV) (Scheme 1) would be conceivable for this, as it forms a larger conjugated π-system, which would lead to an absorbance shift towards the visual spectrum and thus to the observed coloration.

This finding raises the question of why an acetal protection of the aldehyde apparently prevents the ring opening (and thus the BCH), as observed by Kim *et al.* (key point (1) in the introduction),^25^ even though this functionality is not directly involved in the ring-opening mechanism. An answer to this question has already been provided by the computational study of Tashiro *et al.*, which showed that the activation energy of the nucleophilic attack of the hydroxide ion as well as the energy of the resulting intermediate is considerably increased by the acetal protection.^27^ The authors explained this with the loss of π-conjugation of the protected carbonyl functionality, which leads to a more unstable intermediate.

After full conversion of HMF, the yields of the Cannizzaro products remained constant, which confirms their stability in the alkaline solution under standard conditions as reported previously.^26^ Interestingly, in our experiments the product mixture differed from the expected 1 : 1 yield ratio (Fig. 1a). While similar BHMF yields of 12–13% were obtained for all initial HMF concentrations, the final HMFCA yield only lay between 5% (from 20 mM HMF solutions) and 10% (from 100 mM HMF solutions). This finding was confirmed by the UV/Vis spectra, which is explained in more detail in the ESI.†

Since the yield ratio of the Cannizzaro products from HMB was close to 1 : 1, a possible explanation could be that HMFCA is partially incorporated into the forming humic substances, *e.g.* by a reaction with the ring-opened intermediate. This process only seems to occur while HMF is still present in the solution, as the HMFCA yields remained constant after HMF was fully converted. However, it would be expected that this effect is more pronounced at higher HMF concentrations, which contradicts the observation (Fig. 1a). To the best of our knowledge, this effect has not been described yet, as in many published results on alkaline HMF degradation the yields of the Cannizzaro products were not quantified^13,30^ or the obtained HMFCA yield was simply doubled to obtain the total yield of Cannizzaro products.^28^ Krebs *et al.* provided proof of a 1 : 1 yield ratio for 1.0 M HMF solutions in 5 M KOH by quantitative NMR.^26^ Furthermore, they described that the yield of the Cannizzaro products is increased at higher HMF concentrations (up to a limit of 300 mM), without giving yields for the single products. This could be in agreement with our findings, as the effect seems to appear less at higher HMF concentrations. However, the question of why this effect was observed here could not be conclusively answered within this study.

### Evaluation of the UV/Vis spectra during BCH formation

**Fig. 2:**
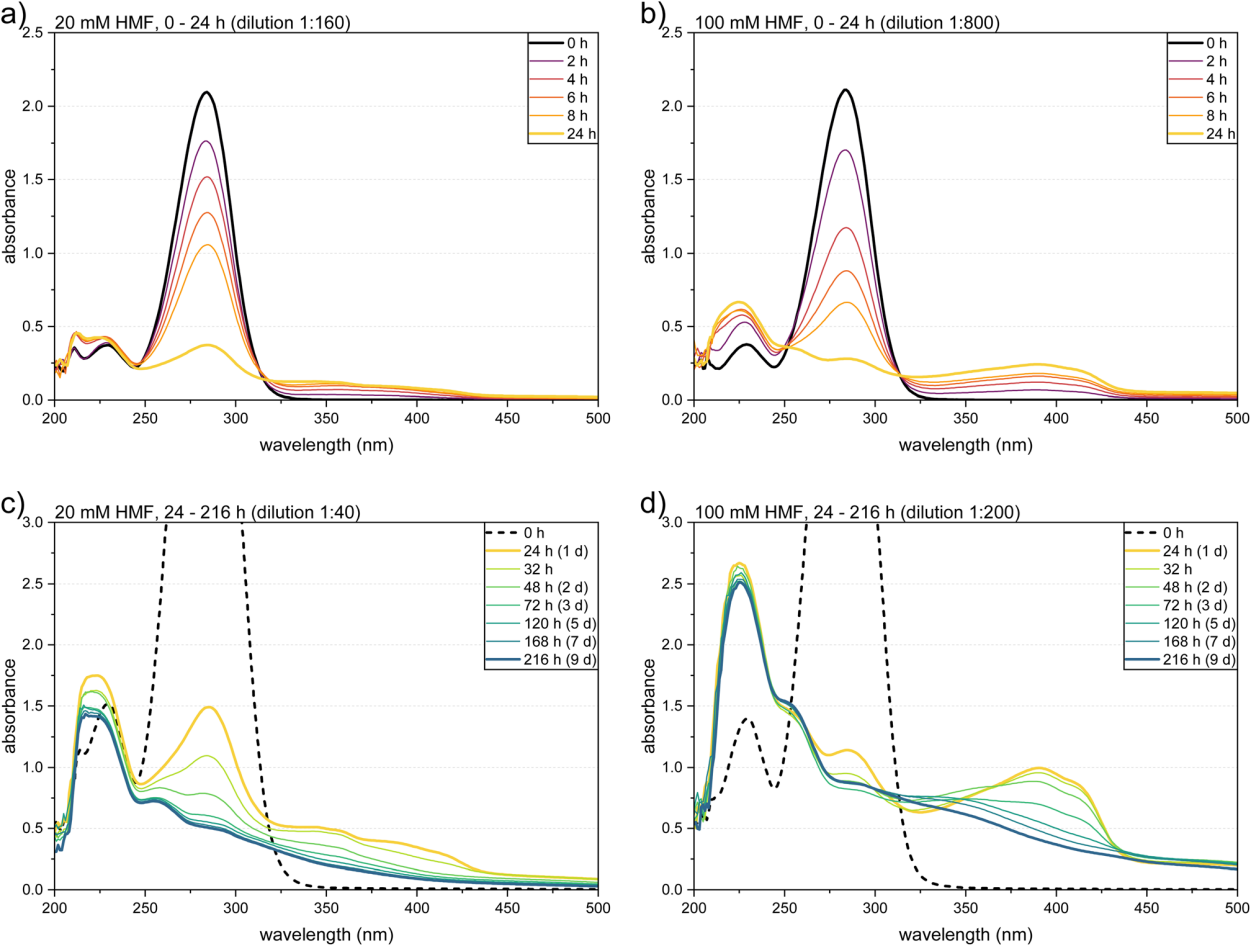
UV/Vis spectra of HMF solutions (20 mM and 100 mM) after specific storing times in 1.0 M KOH: (a) 20 mM and (b) 100 mM HMF after storage of 0–24 h, (c) 20 mM and (d) 100 mM after storage of 24–216 h. The dilution factors were chosen to equalize the initial HMF concentration.

The coloration of the HMF solutions was investigated by UV/Vis spectroscopy. The obtained spectra of the 20 mM and 100 mM HMF solutions are depicted in Fig. 2. To ensure the comparability of the differently concentrated solutions, dilution factors were chosen to equalize the initial HMF concentration and thus the absorbance of the sample. Furthermore, two different dilution factors were measured per sample, to ensure visibility of the less absorbing BCH.

The freshly prepared HMF solutions showed absorption peaks at 230 nm and 284 nm (Fig. 2, black curves). The conversion to the Cannizzaro products was observed by a decrease of the HMF peaks and the formation of two peaks at 223 nm and 251 nm (Fig. 2a and b), which corresponded to BHMF and HMFCA respectively (spectra of the corresponding standards are shown in Fig. S1 in the ESI†). Since the absorbance maxima of the two Cannizzaro products overlapped with the decreasing HMF peaks, they formed a broad absorbance band. Both the decrease of the HMF peaks and the increase of the Cannizzaro peaks were more pronounced at a higher initial HMF concentration. This is in consistence with the higher HMF conversion and HMFCA yield obtained by HPLC (Fig. 1a).

**Fig. 3:**
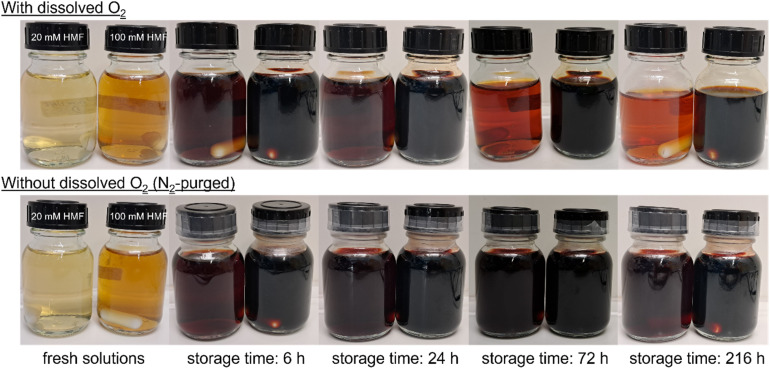
Photographs of HMF solutions in 1.0 M KOH after different storing times in the presence and absence of dissolved O_2_.

The formation of humic material became apparent by the growth of a broad band at longer wavelengths, which extended into the visual range (340–440 nm) and thus caused the observed coloration. The behavior of this band revealed that the BCH formation can generally be divided into two phases. The first phase proceeded within the first 24 h of the storage time, in which most of the HMF was consumed. In this “built-up” phase, the absorbance of the 340–440 nm band increased (Fig. 2a and b), which indicated a growing concentration of oligomeric and polymeric substances with larger conjugated π-systems. The increasing BCH band showed an isosbestic point with the decreasing HMF peak at 314 nm, demonstrating that HMF was stoichiometrically converted to the BCH. Furthermore, the formation of distinct peaks at 391 nm and 412 nm was observed, which became particularly clear in the 100 mM solutions at a low dilution factor (Fig. 2d, yellow curve). This could indicate that certain oligomers are preferentially formed, *e.g.* due to a favored binding position at the ring-opened molecule. However, all solutions had a dark brown color after a storage time of 24 h (Fig. 3).

The second phase started after 24 h, in which a decrease of the 340–440 nm absorbance band was observed (Fig. 2c and d). Especially in the 100 mM solutions, the absorption peaks that had formed during the first phase showed a flattening until the end of the experiment. This caused a visible decolorization of the dark brown solutions (Fig. 3). To the best of our knowledge, this behavior has not been described for HMF yet. It suggests that the formed humic material undergoes an “aging” process, in which the size of the conjugated π-system is reduced again. This is probably caused by reactions of double bonds with dissolved O_2_. Thus, in comparative experiments with N_2_-purged HMF solutions no decolorization effect was observed (Fig. 3) and the BCH peaks at 391 and 412 nm were maintained during 9 days of storage (not shown). As a structural analysis based on the UV/Vis spectra was not possible, further experiments for the determination and classification of the humic structure were carried out, which are described in the next section.

For reasons of comparison, UV/Vis spectra were also recorded during storage of the HMB solutions (Fig. S2 in the ESI†). Here, only the Cannizzaro reaction was observed by the decrease of the HMB absorption peaks (213 nm and 256 nm) and the growth of the HMBA and BHMB peaks at 234 nm and 218 nm, respectively. This confirmed the obtained results from the HPLC measurements.

### Classification and structural analysis of humic substances

During the 9 days of storage time, none of the tested HMF solutions formed a water-insoluble fraction. This is in contradiction with the results of Vuyyuru and Strasser, who reported precipitation of a solid humin fraction already for 5 mM HMF solutions in 0.1 M KOH, however without describing further details.^12^ Thus, the humic material that was formed in our experiments cannot be classified as humins, which, by definition, would be insoluble at any pH.^36^ The other two typical humic compound fractions have a comparatively lower degree of polymerization and can be distinguished by their solubility in acidic solutions, whereby humic acids are insoluble at pH < 2 and fulvic acids are soluble at any pH. Accordingly, humic acids typically have a higher degree of polymerization and thus also a darker color than fulvic acids.^36^

**Fig. 4:**
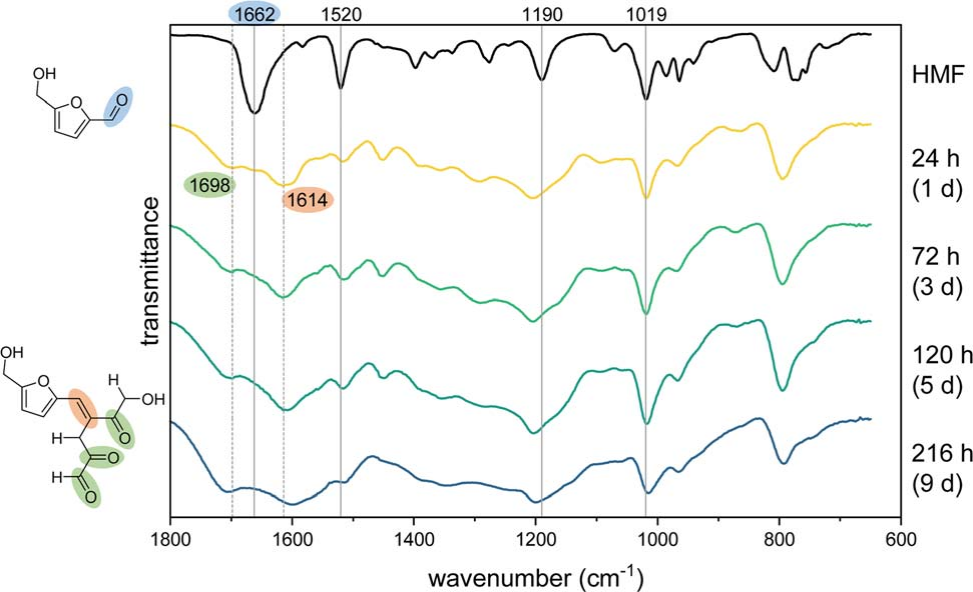
ATR-FTIR spectra of HMF and the solid humic acid fraction after different storing times in 1.0 M KOH. Wavenumbers of characteristic HMF absorption peaks are indicated as straight lines. Newly formed peaks of the humic acids are indicated as dashed lines.

As expected, acidification led to the formation of an insoluble fraction, which, for the 100 mM HMF solutions, increased to approx. 15 wt% of the initial HMF during the storage time. We used this fractionation for further structural characterization of the humic material formed under the presence of dissolved atmospheric O_2_. The separated fulvic acid fraction showed a generally lower absorbance in the UV/Vis spectrum with no remarkable BCH band at 340–440 nm after 24 h (Fig. S3 in the ESI†). This indicated that the decolorization effect is caused by a structural change of the humic acid fraction. Accordingly, we used FTIR spectrometry for a structural analysis of the dried solid after different storing times (Fig. 4).

The spectra of the humic acid fraction showed considerably broader peaks than HMF. Nevertheless, some specific absorption peaks from the HMF spectrum could be found, which allowed a structural characterization. It was also noticeable that the spectra showed great similarity to spectra of acid-catalyzed humic substances (ACH) from HMF, but also from glucose and fructose published by other groups.^21,23,24^ The peaks at 1019 cm^−1^, 1190 cm^−1^ and 1520 cm^−1^ from the HMF spectrum, were also found in the humic acid spectra, albeit slightly shifted. As these wavenumbers correspond to typical values of a ring breathing mode, a C–H in-plane deformation mode and a C=C stretching mode of the furan ring, respectively,^21,37^ these spectra showed that closed furan rings are still present in the humic acid fraction. This also contradicts the statement made by Vuyyuru and Strasser.^12^

Furthermore, the C=O stretching vibration of HMF, which was found at 1662 cm^−1^, was substantially reduced in the humic acid spectra, though new peaks were formed in this spectral region. This indicated the formation of new carbonyl groups through the polymerization. In particular, two new peaks at 1698 and 1614 cm^−1^ were formed. Patil *et al.* also observed the formation of these peaks during the ACH formation and assigned them to the C=O and C=C stretching vibrations of an unsaturated ketone, respectively.^21^ This supports the theory that aldol adducts from HMF and DHH were formed in our case (see structures in Fig. 4).

At longer storage times, the peaks of the humic acid spectra became increasingly broader and thus more unstructured, which indicates the formation of new bonds with deviating vibrational properties. This effect was also apparent for the new peaks at 1698 and 1614 cm^−1^, which additionally showed a shift to higher and lower wavenumbers, respectively. This change illustrates the structural change of the humic acid fraction, which apparently also leads to decolorization. Additional absorbance increases were measured in the wavenumber ranges of 1400–1100 cm^−1^ and 3600–2600 cm^−1^ (not shown), which are in the range of C–O and O–H stretching vibrations and thus indicate the formation of hydroxyl and ether groups. This could be a result of the reaction of C=C double bonds with the dissolved O_2_. A conceivable mechanism for this would be a radical-induced formation of hydroperoxides, similar to the processes that occur during the autoxidation of unsaturated fatty acids such as in linseed oil.^38^ This could lead to new oxygenated functionalities (hydroxide or carbonyl groups) as well as well as cross-linking between different oligomeric molecules.

## Conclusion

**Scheme 2:**
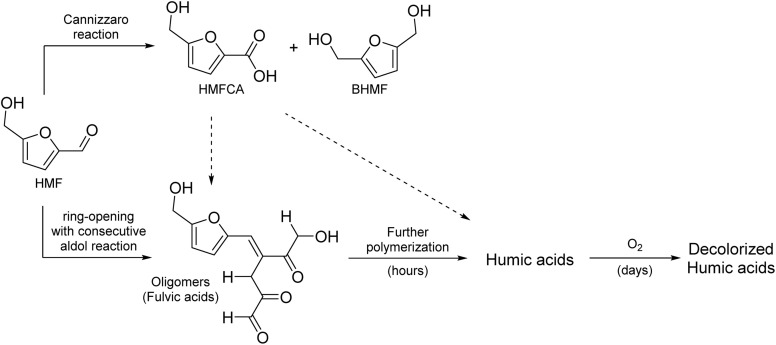
Proposed reaction scheme of the alkaline degradation of HMF under standard conditions.

This study focused on the characterization of the base-catalyzed formation of humic substances from HMF. In the investigated concentration range of 20–100 mM, a complete conversion of the substrate was observed within 3 days, whereby only 18–23% of the initial substance was recovered as Cannizzaro products. Interestingly, the obtained HMFCA yields were consistently lower than those of BHMF. A comparison with 4-(hydroxymethyl)-benzaldehyde showed that, in addition to the Cannizzaro reaction, only a polymerization reaction of HMF involving a ring-opening can take place. It was furthermore observed that the coloration of the HMF solutions only proceeded within the first 24 h of storage (the “build-up” phase), in which a broad absorption band between 340 and 440 nm was formed. This was due to the formation of humic acids, which were shown to be mainly responsible for the coloration. This “build-up” could also involve a reaction with formed HMFCA, which however could not be conclusively clarified in this study. After 24 h, the solutions were subsequently decolorized to a certain extent. This was apparently due to reactions of the humic acid fraction with dissolved atmospheric O_2_. FTIR measurements of this fraction showed that the humic structure became increasingly disorganized and new C=O, C=C, C–O and O–H bonds were formed. Although an explicit structure of the humic acid fraction could not be derived from these spectra, a general reaction scheme for the alkaline HMF degradation under standard conditions can be proposed based on the obtained results (Scheme 2). Further analytical investigations (*e.g.* by LC-MS) could be used to determine exact structures of the formed oligomers and refine this scheme accordingly.

Finally, this study made clear once again that the direct oxidation of HMF under strongly alkaline conditions, which is still preferred for electrochemical and photochemical systems, is not suitable for industrial application. Even a storage time of 1 h led to a mole balance decrease of about 10% due to BCH formation – despite the comparatively low concentrations tested here. Although a solution for this problem cannot be derived from the results of this study, we believe that the better understanding of the humic compound formation obtained here will contribute to the development of concepts for the prevention of this unwanted side reaction and a generally greater awareness by the scientific community. Thus, research should rather focus on the slightly alkaline or even non-alkaline pH region, where only few systems have been published so far.^39–41^ In addition, alternative methods could be developed, such as the prior stabilization of the alkaline HMF solution through the targeted production of Cannizzaro products.^26^

## Experimental section

### Chemicals

The following chemicals were used without further purification: BHMB (99%, abcr), BHMF (99%, AmBeed), HMB (95%, abcr), HMBA (≥98%, abcr), HMF (98%, abcr), HMFCA (98%, TCI), KOH (>85%, Fisher Scientific) and HCl (≥37%, Honeywell). Deionized water was used for the preparation of all solutions.

### Degradation studies

**Scheme 3:**
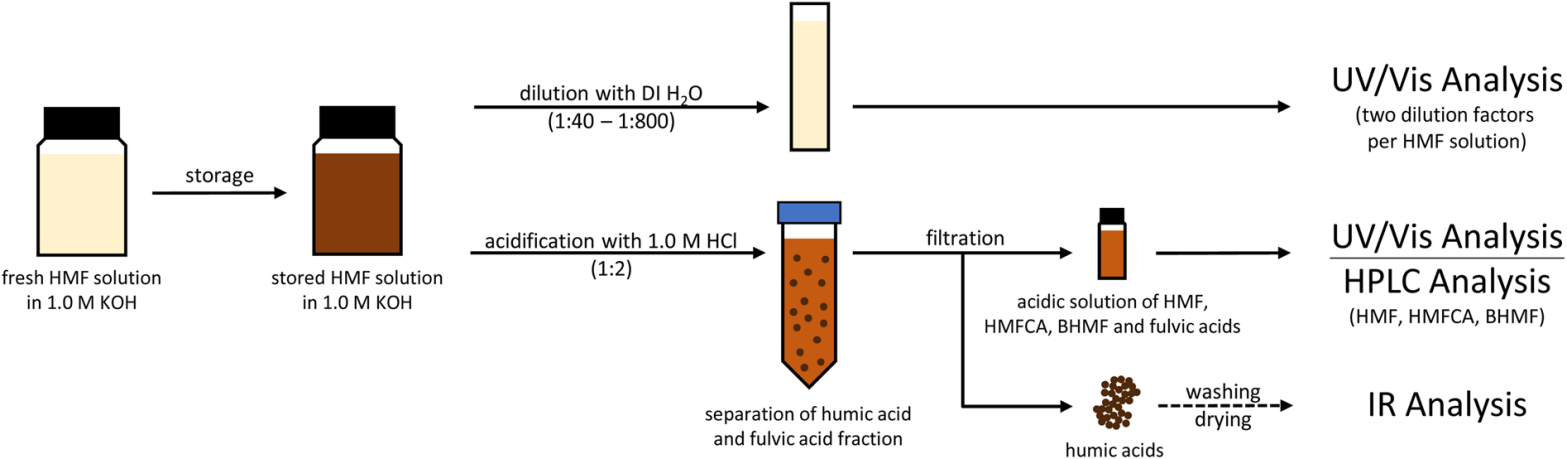
Sample preparation for the used analysis techniques.

For the degradation studies, HMF and HMB solutions with concentrations of 20, 50 and 100 mM were prepared in 1.0 M KOH at 50 mL each. The solutions were stored at 20 °C in closed bottles for 9–13 days and stirred constantly at 500 rpm. To avoid photochemical reactions, the solutions were stored in a blackbox. The experiments were conducted in duplicates. The sample preparation for each analysis method used is graphically shown in Scheme 3. A detailed description can be found in the following sections.

### UV/Vis analysis

For UV/Vis analysis, samples of the HMF and HMB solutions were diluted with DI water to give a total sample volume of 3 mL. The dilution factors were chosen so that they equalize the initial substrate concentration, to obtain comparability between the differently concentrated solutions (Table 1). Two dilution factors were used per sample. DI water was used as a reference. UV/Vis spectra were recorded with a SPECORD 50 spectrometer (Analytik Jena, Germany) at a scan rate of 1 nm s^−1^. The change between the deuterium lamp (UV) and the halogen lamp (Vis) was done at 340 nm. The samples were measured in UV-transparent macro-cuvettes (BRAND, Germany).

**Table 1:** Dilution factors used for UV/Vis analysis and the HPLC analysis

Initial concentration of HMF or HMB	Dilution factors used for UV/Vis analysis	
20 mM	1 : 40	1 : 160
50 mM	1 : 100	1 : 400
100 mM	1 : 200	1 : 800

### HPLC analysis

For concentration analysis of HMF, HMB, their respective Cannizzaro products, LA and FA, 1.0 mL was withdrawn from the respective solution and acidified with 2.0 mL of a 1.0 M HCl solution in order to quench the alkaline reaction. The samples were filtered with syringe filters to remove the insoluble humic acid fraction. Samples were analyzed with a 1260 Infinity II HPLC system (Agilent, USA) using a RESEX ROA Organic Acid column (300 × 7.8 mm, Phenomenex, USA). An aqueous H_2_SO_4_ solution (2.5 mM) was used as eluent with a flow rate of 0.6 mL min^−1^ at 70 °C. The substances were detected with a refractive index detector and a diode array detector. For quantitative analysis, calibration curves were recorded with standard solutions made from the pure substances specified above.

Performance parameters were calculated according to the standard definitions (eqn (S1)–(S3) in the ESI†). The values shown in Fig. 1 represent mean values of the duplicate. Error bars were not depicted for clarity of the figure. Instead, numeric mean values and standard deviations can be found in Table S1 in the ESI.†

### FTIR analysis

To analyze the humic acid fraction, 5 mL of the 100 mM HMF solutions were withdrawn and acidified with 10 mL of 1.0 M HCl. The precipitated humic acid fraction was filtered off, washed with deionized H_2_O, dried in a desiccator for 24 h and weighed. The solid samples were measured in a Spectrum One ATR-FTIR spectrometer (PerkinElmer, USA) as an average of 20 scans. The spectra were baseline-corrected by the automatic correction tool.

## Conflicts of interest

The authors declare no conflict of interest.

## Supplementary Material

RA-015-D5RA02859K-s001

## Data Availability

The data supporting this article have been included as part of the ESI.†
