# Supplementary material for: A mechanistic study on the humic substance formation from 5-(hydroxymethyl)-furfural in alkaline solutions
Source: RSC Adv. 2025 Jul 16;15(31):25132–40. doi: 10.1039/d5ra02859k (PMC12264478; doi:10.1039/d5ra02859k)
Supplement: RA-015-D5RA02859K-s001 [file RA-015-D5RA02859K-s001.pdf]

Supplementary Information (SI) for RSC Advances.
This journal is © The Royal Society of Chemistry 2025

Supporting information

Definition of performance parameters

Eqs. (S1) – (S3) were used to calculate the HMF conversion *X*, the product yield *Y* and the mole balance *MB*. For all equations, a stochiometric coefficient of $\nu = 1$ is assumed.

$$X = \frac{n_{\mathrm{educt,\,0}} - n_{\mathrm{educt,\,t}}}{n_{\mathrm{educt,\,0}}} \cdot 100\ \% \tag{S1}$$

$$Y = \frac{n_{\mathrm{product,\,t}}}{n_{\mathrm{educt,\,0}}} \cdot 100\ \% \tag{S2}$$

$$MB = \frac{n_{\mathrm{educt,\,t}} + \Sigma n_{\mathrm{product,\,t}}}{n_{\mathrm{educt,\,0}}} \cdot 100\ \% \tag{S3}$$

$n_{educt,0}$: amount of educt at the start of the reaction
$n_{educt,t}$: amount of educt at the end of the reaction
$n_{product,t}$: amount of product at the end of the reaction

Additional Figures

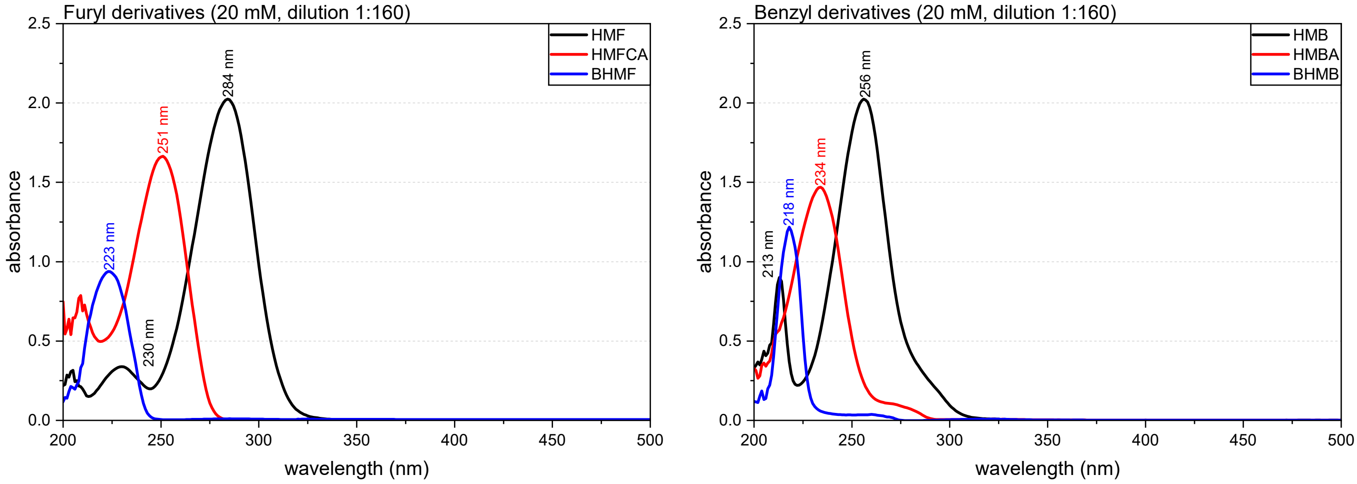

Figure S1: UV/Vis spectra of HMF, HMB and respective Cannizzaro products (20 mM solutions diluted in DI water). Absorbance maxima are indicated.

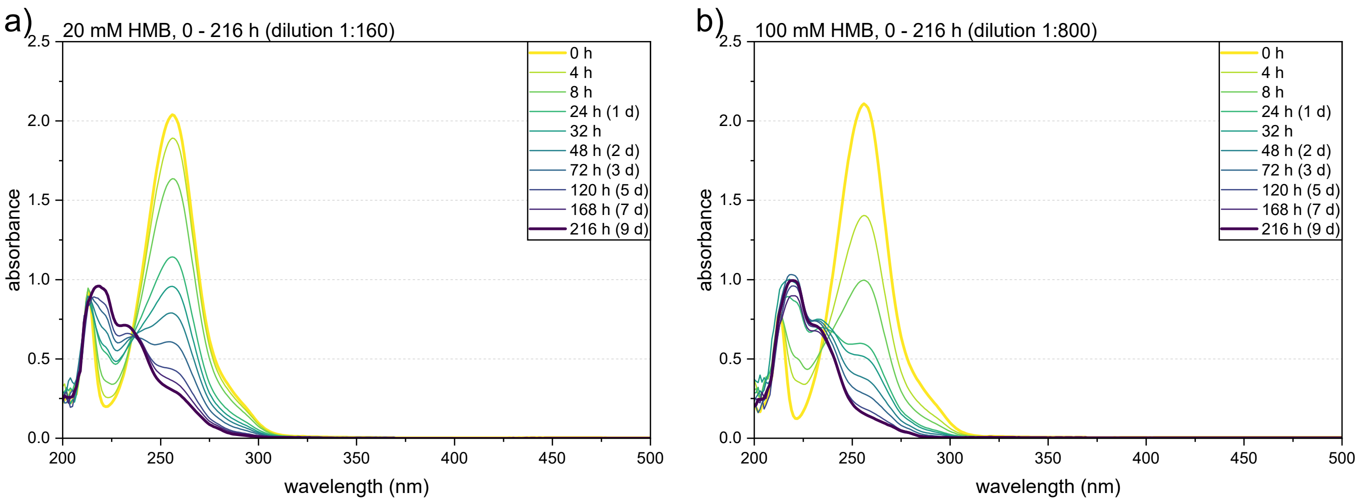

Figure S2: UV/Vis spectra of a) 20 mM and b) 100 mM HMB solutions after specific storing times in 1.0 M KOH (diluted with DI water).

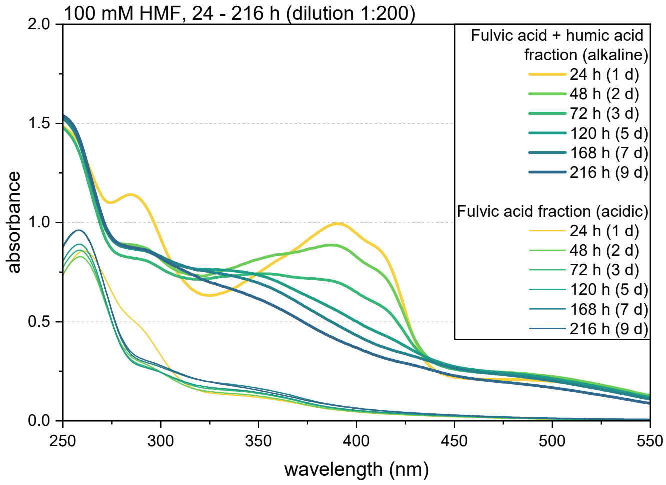

Figure S3: UV/Vis spectra of the alkaline 100 mM HMF solution containing the fulvic acid and humic acid fraction and the acidified solutions only containing the fulvic acid fraction (diluted with DI water).

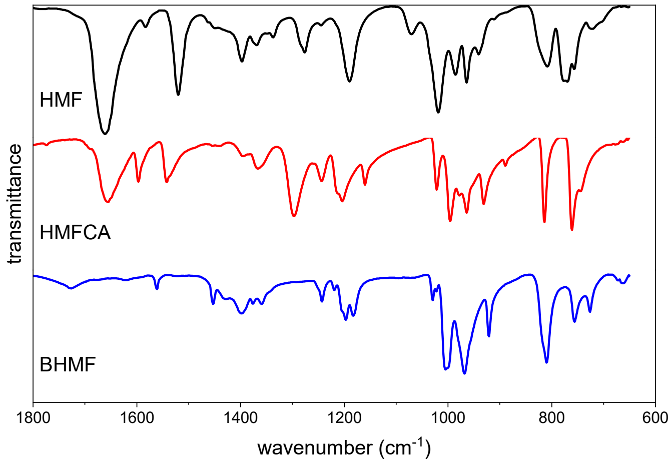

Figure S4: FTIR-ATR spectra of HMF, HMFCA and BHMF as solid standards.

Analysis of the yield ratio of Cannizzaro products from HMF

The HPLC results of the HMF degradation experiments revealed, that the yields of the Cannizzaro products deviated from the expected 1:1 ratio. Here, a greater deviation was determined at lower initial HMF concentrations (Figure 1a). However, an error due to preparation of the HPLC samples could not be ruled out, as only the acid-soluble fraction was analyzed (Scheme S1). It was therefore possible that part of the missing HMFCA was precipitated with the humic acid fraction during the acidification process. To assess this, we used the UV/Vis spectra obtained after 72 h, as these were measured directly from the alkaline solution and HMF was fully converted after this storage time. The spectra of the differently concentrated solutions were normalized against the strongest absorbance to compare the ratio of the HMFCA and BHMF peaks (Figure S5). It was found that comparatively smaller HMFCA peaks were obtained at lower initial HMF concentrations, which corresponds to the HPLC results. Therefore, it is clear that a lower concentration of HMFCA was already present in the alkaline solution and that this is not the result of improper sample preparation.

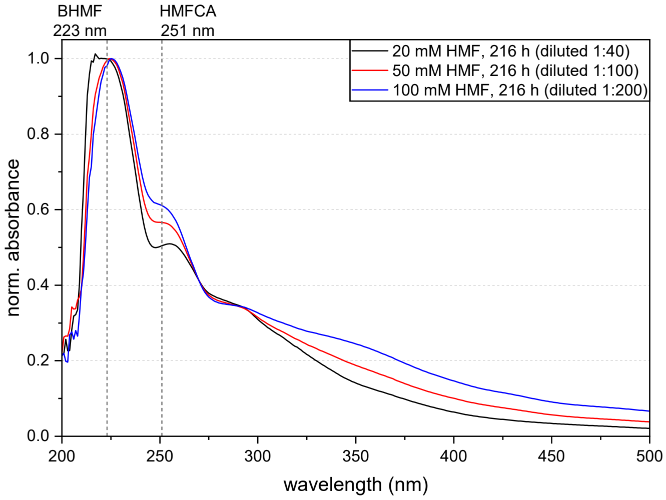

Figure S5: UV/Vis spectra normalized to the highest absorbance of 20 mM, 50 mM and 100 mM after a storage time of 216 h in 1.0 M KOH. The absorbance maxima of BHMF and HMFCA are indicated.

Additional Tables

Table S1: Performance parameters of the degradation experiments of HMF and HMB solutions in 1.0 M KOH under standard conditions. Mean values and standard deviations of duplicate reactions are given.*

	X (%)	*MB* (%)	Y_{HMFCA} (%)	Y_{BHMF} (%)		*X* (%)	*MB* (%)	Y_{HMBA} (%)	Y_{BHMB} (%)
	**20 mM HMF**					**20 mM HMB**			
**1 h**	7.6 ± 0.4	92.4 ± 0.4	n.q.	n.q.		3.0 ± 0.3	97.0 ± 0.3	n.q.	n.q.
**2 h**	16.6 ± 0.1	86.4 ± 0.1	1.3 ± 0.1	1.8 ± 0.0		6.3 ± 0.2	100.0 ± 0.1	3.2 ± 0.1	3.1 ± 0.0
**3 h**	26.0 ± 0.8	78.3 ± 0.8	1.7 ± 0.1	2.6 ± 0.1		10.2 ± 1.1	98.7 ± 1.3	4.5 ± 0.1	4.3 ± 0.1
**4 h**	33.8 ± 0.5	71.5 ± 0.4	2.1 ± 0.1	3.2 ± 0.0		13.0 ± 0.8	98.7 ± 0.6	5.9 ± 0.1	5.8 ± 0.0
**6 h**	46.9 ± 0.8	61.5 ± 0.6	2.8 ± 0.1	5.6 ± 0.1		17.3 ± 0.1	100.0 ± 0.2	8.7 ± 0.2	8.6 ± 0.1
**8 h**	56.7 ± 0.8	53.4 ± 0.6	3.2 ± 0.1	6.9 ± 0.1		22.5 ± 0.1	99.6 ± 0.2	11.2 ± 0.2	10.9 ± 0.1
**10 h**	64.8 ± 0.6	46.7 ± 0.4	3.6 ± 0.1	7.9 ± 0.1		26.6 ± 0.2	99.7 ± 0.3	13.4 ± 0.3	12.9 ± 0.2
**24 h**	90.2 ± 0.2	25.6 ± 0.1	4.7 ± 0.1	11.1 ± 0.0		47.7 ± 0.5	100.2 ± 0.3	24.3 ± 0.1	23.6 ± 0.1
**32 h**	94.7 ± 0.5	21.9 ± 1.4	5.2 ± 0.1	11.4 ± 1.1		55.0 ± 0.3	101.1 ± 0.4	28.6 ± 0.4	27.5 ± 0.3
**48 h**	98.2 ± 0.1	19.9 ± 0.7	5.4 ± 0.1	12.8 ± 0.7		65.1 ± 0.4	101.0 ± 0.0	33.7 ± 0.2	32.3 ± 0.2
**72 h**	100	17.8 ± 0.3	5.2 ± 0.1	12.4 ± 0.2		74.0 ± 0.4	101.1 ± 0.0	38.3 ± 0.2	36.8 ± 0.1
**120 h**	100	17.8 ± 0.1	5.2 ± 0.1	12.6 ± 0.0		83.0 ± 0.2	100.8 ± 0.4	42.3 ± 0.2	41.5 ± 0.3
**168 h**	100	18.0 ± 0.1	5.3 ± 0.1	12.7 ± 0.1		87.4 ± 0.2	102.2 ± 0.1	45.4 ± 0.1	44.1 ± 0.1
**216 h**	100	18.1 ± 0.1	5.4 ± 0.1	12.7 ± 0.1		90.0 ± 0.1	103.2 ± 0.4	47.8 ± 0.3	45.4 ± 0.2
**264 h**	-	-	-	-		92.0 ± 0.0	101.0 ± 1.0	46.7 ± 0.6	46.3 ± 0.5
**312 h**	-	-	-	-		93.2 ± 0.1	101.3 ± 0.0	47.8 ± 0.1	46.7 ± 0.0
	**50 mM HMF**					**50 mM HMB**			
**1 h**	10.4 ± 0.9	92.9 ± 0.9	1.6 ± 0.0	1.7 ± 0.0		8.4 ± 0.9	97.7 ± 1.0	3.1 ± 0.0	3.0 ± 0.0
**2 h**	21.5 ± 0.8	84.1 ± 0.7	2.6 ± 0.0	3.0 ± 0.1		12.9 ± 0.7	98.7 ± 0.7	5.8 ± 0.0	5.7 ± 0.0
**3 h**	33.6 ± 0.5	74.0 ± 0.4	3.5 ± 0.0	4.1 ± 0.1		17.7 ± 0.3	99.2 ± 0.3	8.5 ± 0.0	8.3 ± 0.0
**4 h**	42.2 ± 1.1	67.1 ± 1.0	4.3 ± 0.0	5.0 ± 0.1		22.9 ± 0.2	99.0 ± 0.0	11.1 ± 0.1	10.8 ± 0.1
**6 h**	56.5 ± 1.5	56.2 ± 1.5	5.3 ± 0.0	7.4 ± 0.0		31.8 ± 0.7	98.6 ± 0.8	15.4 ± 0.1	15.1 ± 0.0
**8 h**	66.3 ± 1.4	48.4 ± 1.4	6.0 ± 0.0	8.7 ± 0.0		39.5 ± 0.1	98.0 ± 0.4	19.0 ± 0.3	18.5 ± 0.2
**10 h**	73.3 ± 1.2	42.5 ± 1.3	6.3 ± 0.0	9.5 ± 0.0		44.1 ± 0.3	100.0 ± 0.2	22.3 ± 0.1	21.8 ± ß-ß
**24 h**	94.5 ± 0.5	24.1 ± 0.8	7.4 ± 0.1	11.2 ± 0.2		67.1 ± 0.7	99.4 ± 1.5	33.7 ± 0.4	32.8 ± 0.3
**32 h**	96.7 ± 0.3	22.8 ± 0.8	7.5 ± 0.1	12.0 ± 0.1		72.9 ± 0.2	100.6 ± 0.1	37.1 ± 0.1	36.4 ± 0.1
**48 h**	98.8 ± 0.2	21.8 ± 0.4	7.8 ± 0.1	12.7 ± 0.1		80.3 ± 0.0	100.9 ± 0.4	40.9 ± 0.2	40.3 ± 0.3
**72 h**	99.6 ± 0.0	21.4 ± 0.3	8.1 ± 0.1	12.9 ± 0.2		86.3 ± 0.1	99.7 ± 0.5	43.2 ± 0.2	42.8 ± 0.2
**120 h**	100	20.9 ± 0.2	8.5 ± 0.1	12.5 ± 0.2		91.3 ± 0.2	102.1 ± 1.6	46.5 ± 0.7	46.8 ± 0.7
**168 h**	100	21.4 ± 0.2	8.6 ± 0.1	12.8 ± 0.1		93.8 ± 0.0	100.6 ± 0.4	47.1 ± 0.2	47.3 ± 0.1
**216 h**	100	21.2 ± 0.3	8.5 ± 0.2	12.7 ± 0.2		95.1 ± 0.1	101.7 ± 1.8	48.3 ± 0.9	48.5 ± 0.8
**264 h**	-	-	-	-		96.1 ± 0.0	100.0 ± 0.5	47.8 ± 0.2	48.4 ± 0.2
**312 h**	-	-	-	-		96.7 ± 0.0	100.3 ± 0.3	48.3 ± 0.1	48.7 ± 0.1
	**100 mM HMF**					**100 mM HMB**			
**1 h**	13.6 ± 0.1	91.5 ± 0.1	2.5 ± 0.1	2.5 ± 0.0		10.1 ± 0.1	99.4 ± 0.1	4.8 ± 0.0	4.7 ± 0.0
**2 h**	27.6 ± 0.7	81.2 ± 0.8	4.3 ± 0.1	4.6 ± 0.0		19.3 ± 0.2	98.5 ± 0.2	9.0 ± 0.0	8.8 ± 0.0
**3 h**	39.9 ± 0.3	71.9 ± 0.3	5.6 ± 0.1	6.2 ± 0.1		26.8 ± 0.1	98.5 ± 0.1	12.7 ± 0.0	12.5 ± 0.0
**4 h**	50.7 ± 0.5	62.9 ± 0.4	6.5 ± 0.0	7.1 ± 0.1		33.4 ± 0.0	98.2 ± 0.0	16.0 ± 0.0	15.7 ± 0.0
**6 h**	65.6 ± 0.8	50.9 ± 0.8	7.9 ± 0.1	8.6 ± 0.1		43.5 ± 0.1	98.8 ± 0.1	21.3 ± 0.0	20.9 ± 0.1
**8 h**	74.9 ± 0.6	43.0 ± 0.6	8.5 ± 0.1	9.5 ± 0.1		52.1 ± 0.8	97.1 ± 1.4	24.9 ± 0.3	24.4 ± 0.3
**10 h**	80.7 ± 0.5	38.1 ± 0.4	8.8 ± 0.1	10.0 ± 0.2		57.1 ± 0.1	99.5 ± 0.1	28.6 ± 0.0	28.0 ± 0.0
**24 h**	96.4 ± 0.2	23.5 ± 0.5	9.3 ± 0.2	10.6 ± 0.1		77.1 ± 0.1	99.6 ± 0.2	38.6 ± 0.1	38.2 ± 0.0
**32 h**	97.8 ± 0.1	22.8 ± 0.2	9.5 ± 0.1	11.1 ± 0.0		82.0 ± 0.0	99.5 ± 0.0	40.8 ± 0.0	40.6 ± 0.0
**48 h**	99.2 ± 0.0	22.7 ± 0.0	9.7 ± 0.1	12.2 ± 0.1		87.4 ± 0.0	99.6 ± 0.1	43.5 ± 0.0	43.5 ± 0.1
**72 h**	99.5 ± 0.0	23.0 ± 0.0	9.9 ± 0.1	12.6 ± 0.1		91.3 ± 0.1	99.7 ± 0.5	45.4 ± 0.2	45.7 ± 0.2
**120 h**	100	21.5 ± 0.3	10.0 ± 0.1	11.5 ± 0.2		94.7 ± 0.0	100.0 ± 0.6	47.0 ± 0.3	47.7 ± 0.3
**168 h**	100	22.0 ± 0.1	10.2 ± 0.1	11.7 ± 0.0		96.2 ± 0.0	99.7 ± 0.1	47.6 ± 0.0	48.3 ± 0.0
**216 h**	100	22.6 ± 0.2	10.5 ± 0.1	12.1 ± 0.1		97.1 ± 0.0	100.1 ± 0.2	48.3 ± 0.1	48.9 ± 0.1
**264 h**	-	-	-	-		97.7 ± 0.0	99.1 ± 0.2	47.8 ± 0.1	48.9 ± 0.1
**312 h**	-	-	-	-		98.0 ± 0.0	99.7 ± 0.4	48.3 ± 0.2	49.3 ± 0.2

n.q. = not quantifiable

* Although standard deviations should generally only be used for at least three replicates, we chose these values for a better comparability with the respective reaction performance data.
